# Correlation of the oral health and biochemical profile in hemodialysis patients with end-stage renal diseases

**DOI:** 10.1007/s10266-025-01071-y

**Published:** 2025-03-17

**Authors:** Hosam Nagy, Wafaa Saleh, Ghada El Kannishy, Jilan Mohamed Youssef

**Affiliations:** 1 Oral Medicine, Periodontology, Diagnosis and Oral Radiology Department, Faculty of Dentistry, Mansoura University, Mansoura City, 33516 Egypt; 2 Internal Medicine Department, Faculty of Medicine, Mansoura University, Mansoura, Egypt

**Keywords:** Hemodialysis, End-stage renal diseases, Oral health

## Abstract

The current study aims to investigate the oral health of end-stage renal diseases (ESRD) patients undergoing hemodialysis and to correlate it with the biochemical profile of the patients. The current study included 43 ESRD patients on regular hemodialysis. The oral hygiene status, dental, and periodontal health were measured by the following indices: decayed, missed, and filled teeth (DMFT) index, visible plaque index (VPI), and Russell periodontal index (RPI). Several biochemical, hormonal, and hematological parameters were evaluated for each patient. The correlation of the oral health status with the biochemical profile of the patients was measured. The mean age of the participants in the current study was (51.67 ± 14.7) years. About 81% of the participants were hypertensive (35), 19% were diabetic (8), and 28% showed HCV positivity (12). VPI showed 30% of patients had fair oral hygiene and 44% had poor oral hygiene. According to RPI, periodontitis was detected in more than 95% of patients. It was significantly correlated with the age and body mass index (BMI) of the patients. DMFT index was significantly positively correlated to RPI and serum transferrin saturation. RPI scores showed a significant correlation between the ages of the patients and their BMI. VPI significantly correlated with serum calcium, but not with other biochemical variables. ESRD patients treated with hemodialysis showed several oral health concerns. Most of these patients had periodontitis, which was more frequent in diabetics and elderly patients. Advanced grades of periodontitis were associated with older age and increased BMI.

## Introduction

Chronic renal failure (CRF) is defined as an advanced decline and irreversible loss of renal functions associated with a reduced glomerular filtration rate (GFR). The prevalence rate of CRF is difficult to measure due to the asymptomatic nature of early to moderate CRF [1]. Patients are considered to have chronic kidney disease (CKD) if the GFR is less than 60 ml/min per 1.73 m^2^ associated with continuous signs of renal damage that last at least 3 months. CKD may progress to end-stage renal disease (ESRD) if the GFR falls below 15 ml/min per 1.73 m^2^ in which the kidney function has deteriorated to the point where it can no longer sustain life for a long period, necessitating treatment with either dialysis or renal transplantation [2, 3].

The beak of ESRD shows a bimodal age distribution. The first peak is mostly detected in patients with an age range of 20–40 years and is mostly associated with CKD of unknown etiology (73%). The second peak occurs between 50 and 70 years and is commonly detected in diabetic and hypertensive patients [4, 5].

The well-established risk factors for CKDs include cardiovascular diseases, uncontrolled diabetes, hypertension, and older age. These factors can contribute to the pathogenesis of CKD and result in aggravation of nephron loss, perpetuating the cycles of injury and ultimately resulting in ESRDs. Similarly, many of these conditions have been linked to oral health problems [6–8].

Numerous oral diseases have been detected in patients with renal disorders including gingival enlargement, xerostomia, bad taste, oral infections, oral hairy leukoplakia, uremic stomatitis, dental anomalies, and bone lesions [6]. The oral changes in CKD patients may be attributed to altered immune response, systemic inflammation, and changes in biochemical markers. The association between CKD and periodontal diseases may be attributed to many factors, the most important is the state of uremia in CKD patients which leads to an altered immune system due to impaired function of B- and T lymphocytes as well as monocytes and macrophages resulting in decreased host response to Gram-negative bacteria [9]. In addition, poor oral hygiene is common in CKD patients, probably due to the psychological burden of the disease and its therapy. Furthermore, uremia was associated with increased severity and prevalence of periodontitis in dialysis patients [6, 10].

In addition, oral health was affected by CKD, while oral findings can play a role in the diagnosis of renal diseases. Increased plaque accumulation and higher prevalence and severity of periodontal diseases were commonly observed in CKD patients [11, 12]. Periodontal diseases are inflammation and infection of the periodontium supporting the tooth. The primary cause of periodontal diseases is the accumulation of bacterial biofilm on the teeth and gingiva that stimulate the inflammatory response resulting in tissue destruction. [13–15] Different surgical and non-surgical treatment modalities were utilized for periodontal diseases, while the treatment depends on the severity of the disease and the patient’s response [16–19].

The association between periodontal diseases and several systemic diseases including diabetes mellitus, cardiovascular diseases, pulmonary diseases, and others have been reported [20–22]. Most recently, a significant positive association between periodontitis and older CKD patients was established [23].

Despite the wide range of oral diseases detected in patients with ESRD, dental visits are infrequent, with minimum awareness of the problem [24, 25]. To our knowledge, very few studies have shed strong light on the relation between dental and periodontal diseases and the biochemical, hormonal, and hematological alterations in hemodialysis patients [6, 11, 26]. Thus, it is imperative that further studies are required for the precise identification of dental and periodontal diseases in ESRD patients and their biochemical correlates in our locality. The present study aims to identify the frequency and severity of oral diseases in patients suffering from ESRD undergoing regular hemodialysis and to explore its clinical and biochemical associations.

## Methods

We conducted a cross-sectional study on 43 patients with ESRD undergoing regular hemodialysis. The participants of the current study were collected from the Nephrology and Dialysis Unit at Mansoura University. The study proposal was approved by the Ethical Committee at the Faculty of Dentistry, Mansoura University (IRB Approval Number: A07090419) and all research procedures were performed in accordance with relevant guidelines of this committee. All patients signed an informed consent of approval, and all the procedures were explained to the patients before conducting the study. Patient’s data were kept private and confidential. The current study was conducted between January 2020 and March 2022 and it was performed in accordance with the Declaration of Helsinki.

The periodontal and dental examinations were performed by direct interviewing the ESRD patients, in addition to using medical records for the collection of patients’ medical data. Blood samples were collected from all the included cases to correlate the oral and medical findings with the biochemical findings.

### Measuring the clinical outcomes

The clinical outcomes were measured by two independent examiners (HN, JMY). The inter-examiner reliability test revealed an agreement of 86.1%, with Cohen's kappa coefficient (k) calculated at 0.64, indicating a moderate to substantial agreement.

### Patients’ selection

The following inclusion and exclusion criteria were executed in the current study. Inclusion criteria included patients with ESRD undergoing regular hemodialysis who are ≥ 15 years old and undergoing regular hemodialysis for at least 6 months and patients with enough teeth to allow accurate evaluation of the visual plaque index (VPI) and other periodontal assessments.

We applied the following exclusion criteria during selection of the participants: completely edentulous patients, patients with significant missing teeth that precluded proper VPI examination, patients with malignant tumors, patients with a history of kidney transplantation, patients with decompensated cardiac, pulmonary, or hepatic failure, patients with contagious diseases such as HIV, and patients who refused to sign a written consent for approval for conducting the study.

### Demographics of patients

All the following socio-demographic data were recorded for the included patients: age, gender, marital status, and current smoking habit.

### Medical history

A thorough medical history was obtained from the medical records with particular emphasis on diabetes mellitus, hypertension, cardiovascular diseases, hepatic diseases, autoimmune disorders, and nutritional status. Different medications utilized by hemodialysis patients were noted and observed. In addition, the history of parathyroidectomy was recorded. Body weight and body mass index (BMI) were recorded. Duration of dialysis and *KT*/*V* (a measurement for efficiency of dialysis) were noted. *KT*/*V* refers to *K* = clearance—the amount of urea that the dialyzer can remove (liters/minute), *t* = time—the duration of treatment (minutes), and volume—the amount of body fluid (*l*). Mortality was observed during the duration of the study. Malnutritional status was measured using malnutrition inflammation scoring (MIS).

### Dental and periodontal examinations

The intraoral examination was visually performed under a good source of light with a dental probe and mouth mirror. The patients were examined before the dialysis procedures. All the patients were asked to stop brushing their teeth prior to the examination. Then, the teeth were dried with air. Oral and dental examinations were performed using the following indices: decayed, missed, and filled teeth (DMFT) index, visible plaque index (VPI), and Russell periodontal index (RPI).

### Decayed, missed, and filled teeth (DMFT) index [27, 28]

We used the DMFT index to measure the prevalence of dental caries among the patients of our study. D stands for decayed teeth, M stands for missing teeth due to caries, and F stands for filled teeth.

### Visible plaque index (VPI) [29]

This index was used to assess the thickness of the debris at the cervical margins of the tooth near the gingival margin. Four areas were examined for each tooth: labial, lingual, mesial, and distal. Six teeth were examined which are: 16, 12, 24, 36, 32, and 44. Each tooth is examined for four surfaces and scored according to the following criteria:

**0**: No plaque.

**1:** A thin film of plaque adheres to the free gingival margin.

**2:** Moderate accumulation of soft deposits in the free gingival margin and inside pockets, but not on interdental spaces.

**3:** Abundant accumulation of soft deposits in free gingival margins and inside pockets and on different tooth surfaces including interdental spaces.

The scores for four surfaces were calculated and summation is made then divided by 4. The VPI index is calculated by summation of scores of six tested teeth and then divided by 6.

If one or more of the index teeth were missing, we substituted them with the adjacent or similar teeth to preserve the accuracy of the assessment. If we found that there was no suitable substitute tooth available, we noted the missing tooth, and the calculation was adjusted accordingly.

### Four ratings are possible in the calculation

0: excellent oral hygiene,

0.1–0.9: good oral hygiene,

1–1.9: fair oral hygiene,

2–3: poor oral hygiene.

### Russell periodontal Index (RPI) [30]

RPI is used to measure periodontal diseases. According to this index, each tooth is examined and gets a score (0, 1, 2, 6, 8). Scoring for each tooth was added and the total was divided by the number of teeth examined.

### Interpretation of rating

0–0.2 = normal.

0.3–0.9 = simple gingivitis.

0.9–1.9 = beginning destructive periodontal disease.

2–4.9 = established periodontal disease.

5–8 = terminal periodontal disease.

### Scoring criteria

**0:** no signs of inflammation.

**1:** simple gingivitis.

**2:** established gingivitis all around the tooth, but no loss in the epithelial attachment.

**6:** gingivitis with pocket formation.

**8:** advanced periodontal destruction with loss of masticatory function (tooth can be loose).

Scores of individual teeth are added and divided by number of teeth examined.

To accurately define the severity and/or extent of the dental and periodontal diseases, certain indices were collected by applying some calculations on certain dental and periodontal observations. The DMFT index was calculated as the summation of total decayed, missed, and filled teeth for each patient. The VPI was calculated as the measured amount of dental plaque on the cervical part of six index teeth using visual examination with the assistance of a dental probe. In addition, with the assistance of a graduated periodontal probe, the RPI index, averaged on all teeth depending on the severity of inflammation and depth of the pockets, was calculated.

The periodontal health status of the studied patients was categorized as chronic gingivitis, localized chronic periodontitis, and generalized chronic periodontitis. According to another classification of the extent of periodontal disease [31], chronic gingivitis (gingival inflammation without pocket formation), localized chronic periodontitis (presence of periodontal pocket and bone loss in less than one-third of the examined teeth), and generalized chronic periodontitis (presence of periodontal pockets and bone loss in more than one-third of the teeth).

### Laboratory measurements

Routine laboratory tests for hemodialysis patients included complete blood count (CBC), serum creatinine, and serum levels of calcium, phosphorus, parathyroid hormone (PTH), albumin, total cholesterol, and total protein. In addition, plasma pH, serum bicarbonate, and iron status (serum iron, serum total iron-binding capacity, transferrin saturation) were recorded. Moreover, serum ferritin, highly sensitive C-reactive protein (hsCRP), erythrocyte sedimentation rate (ESR), and interleukin 6 (IL-6) were also measured as markers of inflammation.

### Blood sample collection

Blood samples were collected by the nursing team at Mansoura University Nephrology and Dialysis Unit. Venous blood was withdrawn from the antecubital vein by using a standard venipuncture method. The obtained blood samples were collected in vacutainer tubes and anticoagulated with ethylenediaminetetraacetic acid (EDTA).

### High-sensitive C-reactive protein (hs-CRP)

hs-CRP was measured using enzyme-linked immunosorbent assay (ELISA) which was made by Chemux Bio Science Kit. This was performed by using monoclonal antibodies directed toward specific antigens on CRP molecules. This monoclonal anti-CRP antibody was used for solid immobilization. The antibody–enzyme conjugate solution is a goat anti-CRP antibody. The prepared sample was left to react with the two antibodies, resulting in the CRP molecules being packed between the solid phase and enzyme-linked antibodies after a 45-min incubation period at room temperature. A reagent tetramethylbenzidine (TMB) was added and then incubated for 20 min resulting in the appearance of a blue color which was changed later to yellow. The concentration of CRP was directly proportional to the color intensity of the sample in the test. Spectrophotometric absorption was measured at 450 nm.

### Interleukin-6 (IL-6)

Measurement of IL-6 was performed by EIAab Human Interleukin-6 (IL-6) ELISA Kit. A plate was coated with antibodies against the target antigen. Samples were added to plate wells with antibodies (conjugated to biotin) specific to the target antigen. Horseradish peroxidase (conjugated to avidin) was added to each plate well and incubated. In each well, the TMB substrate was added. The color change was noticed only in wells containing target antigen and enzyme-conjugated avidin and biotin-conjugated antibody. Sulfuric acid was added to terminate the enzyme–substrate reaction. Spectrophotometric color change at wavelength 450 ± 2 nm was recorded.

### Measuring parathyroid hormone, iron parameters, and ferritin

Parathyroid hormone (PTH) was measured by radioimmune assay test using specific antiserum (GP-1) directed against two terminal parts in PTH which are amino carboxyl parts. Serum iron and total iron-binding capacity (TIBC) were measured using the Elitech kit. TSAT = serum iron divided by TIBC. Human ferritin ELISA kits were used to measure serum ferritin.

### Statistical analysis

We used the Statistical Package for Social Science (SPSS) program version 24 to perform the statistical analysis of the current study. Continuous data were expressed as means ± standard deviations (SD) for normally distributed variables or as medians and interquartile ranges for the non-parametric variables. Nominal data were summarized as numbers and percentages. To compare the two samples of non-normally distributed variables, we used the “Mann–Whitney test”, while t test was used for comparing the parametric data. Numerical correlations were analyzed using Spearman’s correlation coefficient. The significance level was set at p ≤ 0.05 for all tests.

## Results

The current study included 24 males (55%) and 19 females (45%) with mean age and standard deviation 51.67 ± 14.7 years, and their mean dialysis duration was 45 ± 37.7 months. The demographic data and medical conditions of the included cases are presented in Table 1. The biochemical data of the participants are presented in Table 2.

**Table 1:** Demographic and medical characteristics of participants undergoing hemodialysis

	Males	Females	*p* value	Total number
No. of participants	24	19		43
Age (years) (mean ± SD)	51.87 + 11.7	50.68 + 17.85	0.4	
Mean hemodialysis duration ± SD (months)	49.95 + 12.3	38.77 + 9.87	0.17	41
Mean body mass index score ± SD (kg/m^2^)	28.68 + 4.87	28.84 + 5.38	0.45	41
Current smokers	5	0	0.15	5
Diabetes mellitus	5	3	0.31	8
HCV	7	5	0.40	12
Hypertension	21	14	0.39	35
History of parathyroidectomy	0	2	0.15	2
Mortality	9	4	0.3	13

**Table 2:** Description of the biochemical data among the studied patients

Laboratory parameters	Outcomes		Normal range
Serum creatinine (mg/dL)	Mean ± SD	10.98 ± 3.2	0.6–1.3 mg/dL
Serum calcium (mg/dL)	Mean ± SD	8.37 ± 0.83	8.5–10.5 mg/dL
Serum phosphorus (mg/dL)	Mean ± SD	4.74 ± 1.63	2.5–4.5 mg/dL
Serum parathyroid hormone (pg/mL)	Median (Q1–Q3)	530 (227.0–958.5)	10–65 pg/mL
Serum albumin (g/dL)	Median (Q1–Q3)	4 (3.80–4.18)	3.5–5.0 g/dL
Serum total protein (g/dL)	Mean ± SD	6.97 ± 0.28	6.0–8.3 g/dL
Serum cholesterol (mg/dL)	Mean ± SD	151.3 ± 47.07	< 200 mg/dL
pH	Median (Q1–Q3)	7.37 (7.34–7.39)	7.35–7.45
Serum bicarbonate (mmol/L)	Mean ± SD	20.79 ± 2.09	22–28 mmol/L
Serumi (mcg/dL) *	Median (Q1–Q3)	83 (67.0–96.00)	60–170 mcg/dL
Hemoglobin (g/dL)	Mean ± SD	10.28 ± 1.55	Males: 13.5–17.5 g/dL Females: 12.0–15.5 g/dL
White blood cells (WBCs, × 10^9^/L)	Mean ± SD	5.82 ± 1.8	4.0–11.0 × 10⁹/L
Neutrophils (× 10^9^/L)	Median (Q1–Q3)	3.2 (2.80–3.80)	2.0–7.5 × 10⁹/L
Lymphocytes (× 10^9^/L)	Mean ± SD	1.62 ± 0.6	1.0–3.0 × 10⁹/L
Erythrocyte sedimentation rate (ESR, mm)	Median (Q1–Q3)	30 (10.0–45.0)	0–20 mm/hour
High sensitive C-reactive protein	Median (Q1–Q3)	11,350 (4800–15500)	< 3.0 mg/L
Interkeukin 6 (IL-6, pg/mL, normally)	Median (Q1–Q3)	4.15 (3.33–4.80)	0–43.5 pg/mL
Malnutrition inflammation score (MIS)	Median (Q1–Q3)	4 (3.00–6.50)	–

### Dental and periodontal examinations

#### The DMFT index

The investigation of DMFT index scores among the patients showed that more than one-fourth of the patients had complete sets of teeth, while about 16% of patients had ≥ 16 missing teeth. Detection of tooth decay revealed that more than 53% of the patients had no tooth decay. None of the participants had more than four filled teeth, while 79% of the patients had no tooth filling, irrespective of having decay or not. It was noticeable that tooth filling was equally distributed between both males and females, while those with tooth filling were significantly younger (Table 3).

**Table 3:** Distribution of the DMFT index data among the study patients

DMFT	Number of teeth	Number of patients	%
Number of decayed teeth	0	23	53.5
	1	6	14
	2	5	11.6
	3	3	7
	4	3	7
	5	2	4.7
	≥ 11	1	2.3
Number of missed teeth	0	11	25.6
	1–5	15	34.9
	6–15	10	23.3
	≥ 16	7	16.3
Number of filled teeth	0	34	79.1
	1	4	9.3
	2	4	9.3
	4	1	2.3
Total number of patients	43		

#### VPI of the studied patients

Measurement of the VPI of the cases showed that less than 10% of the studied patients had no dental plaques (score 0), while less than one-third of them had a score of two or more (poor oral hygiene).

### RPI scores of the studied patients

Screening the periodontal health status among the included patients was performed using the RPI. Healthy periodontium and simple gingivitis were detected in one patient from each group (2.3%). Periodontitis was detected in more than 95% of patients Table 4.

**Table 4:** Distribution of the grades of dental and periodontal indices among the studied patients

	Numbers of patients	%
DMFT score grade subgroups		
Low caries group (scores ≤ 1)	15	34.9
Moderate caries group (scores > 1: < 5)	13	30.2
High caries group (scores ≥ 5)	15	34.9
Grading of VPI		
Excellent oral hygiene (scores 0)	4	9.3
Good oral hygiene (scores > 0: < 1)	8	18.6
Fair oral hygiene (scores ≥ 1: < 2)	12	27.9
Poor oral hygiene (scores ≥ 2)	19	44.2
Grades of RPI		
Normal (scores ≤ 0.2)	1	2.3
Simple gingivitis (scores > 0.2: < 1)	1	2.3
Beginning periodontitis (scores ≥ 1: < 2)	7	16.3
Established periodontitis (scores ≥ 2: < 5)	25	58.1
Terminal periodontitis (scores ≥ 5)	9	20.9

To facilitate the comparisons between the different grades of the indices, each of the three indices were classified into few subgroups, according to the extent or severity of the observations. Thus, the DMFT index was divided into three subgroups depending on the level of the scores calculated; the VPI was divided into four categories according to Loe and Silness [29] depending on the average score of the examined patient where the scores reflect the level of oral hygiene; and the RPI index was divided into four subgroups according to the severity of the scores described by Russel [30] (Table 4).

### Comparison of the oral indices, demographic data, and medical profile of the participants

The scores of the three studied indices were compared according to gender, presence of diabetes, hypertension, and HCV antibody positivity, and other clinical and prognostic classifiers. Patients with HCV antibody positivity had significantly higher scores of both the DMFT and RPI indices. There were no significant differences in any of the studied indices between the patients of both genders, those with hypertension, those with higher malnutrition inflammation scores, and those who died during the observation period of the study. On the other hand, patients suffering from diabetes mellitus had significantly lower VPI scores (Table 5).

**Table 5:** Relation between gender, morbidity, and mortality categories and the studied dental scores

Dental score		DMFT scores		VPI scores		RPI scores	
Classifier	Class	Mean ± SD or median (Q1–Q3)	*p* value	Mean ± SD or Median (Q1-Q3)	*p* value	Mean ± SD or median (Q1–Q3)	*p*P value
Gender	Males (*n* = 24)	3.0 (1.0–9.0)	0.710	1.60 ± 1.00	0.929	3.33 ± 1.64	0.297
	Females (*n* = 19)	3.0 (1.0–5.0)		1.63 ± 0.89		2.83 ± 1.40	
Diabetes mellitus	Yes (*n* = 8)	2.0 (1.0–7.0)	0.766	0.88 ± 1.04	0.01*	3.17 ± 2.02	0.847
	No (*n* = 33)	3.0 (1.0–7.5)		1.78 ± 0.83		3.05 ± 1.40	
Hypertension	Yes (*n* = 35)	3.0 (1.0–8.0)	0.642	1.60(0.60–2.40)	0.653	3.12 ± 1.59	0.666
	No (*n* = 6)	3.0 (2.0–5.5)		1.83(1.20–2.25)		2.83 ± 0.98	
Hepatitis C virus	Yes (*n* = 12)	6.0 (1.5–13.5)	0.024*	1.37 ± 1.04	0.296	4.86(2.25–5.75)	0.014*
	No (*n* = 29)	2.0 (0.5–5.0)		1.71 ± 0.88		2.59(1.96–3.29)	
Malnutrition Inflammation score	Low ≤ 4 (*n* = 19)	4.0 (1.0–8.0)	0.636	1.80 ± 0.93	0.402	3.35 ± 1.54	0.364
	High > 4 (*n* = 18)	2.5 (1.0–6.0)		1.54 ± 0.92		2.90 ± 1.41	
Mortality	Living (*n* = 29)	2.0 (0.5–5.5)	0.147	1.61 ± 0.95	0.949	3.14(2.20–4.29)	0.337
	Died (*n* = 14)	4.0 (1.75–10.5)		1.63 ± 0.97		2.14(1.79–4.41)	

### Periodontitis and biochemical parameters

The mean age of patients with generalized chronic periodontitis was statistically significantly higher than the corresponding values of patients with the other two classes of extent of periodontitis. Similarly, the mean value of BMI was higher in generalized chronic periodontitis compared to the corresponding value of patients with chronic gingivitis. We detected a statistically significant difference in blood hemoglobin levels between the three groups of periodontal diseases. Apart from that, all the other biochemical, hematological, and inflammatory variables were insignificantly different in the generalized periodontitis group compared to the other two subgroups (Table 6).

**Table 6:** Comparison of the observed subgroups of RPI regarding some clinical and biochemical data

		Chronic gingivitis	Localized periodontitis	Generalized periodontitis	*p* value
Age (years)	*N*	10	11	22	< 0.001*
	Mean ± SD	35.1 ± 10.7	48. 5 ± 14.6	60.2 ± 9.2	
BMI (kg/m^2^)	*N*	9	10	18	0.012*
	Mean ± SD	24.4 ± 4.4	29.1 ± 4.8	30.2 ± 4.5	
Dialysis duration (months)	*N*	10	11	20	0.725
	Median (Q1–Q3)	25.0 (11.5–77.0)	46.0 (20.0–52.0)	34.0 (15.0–69.5)	
KT/V	*N*	10	11	20	0.247
	Mean ± SD	1.42 ± 0.34	1.32 ± 0.32	1.21 ± 0.32	
S. Albumin (g/dL)	*N*	8	10	18	0.077
	Mean ± SD	3.7 ± 0.45	4.0 ± 0.23	4.0 ± 0.26	
S. Cholesterol (mg/dL)	*N*	9	10	18	0.195
	Mean ± SD	147.8 ± 43.8	173.9 ± 57.7	140.5 ± 40.0	
S. Calcium (mg/dL)	*N*	10	11	20	0.447
	Mean ± SD	8.4 ± 0.79	8.6 ± 0.62	8.2 ± 0.96	
S. Phosphorus (mg/dL)	*N*	10	11	20	0.841
	Mean ± SD	5.0 ± 1.9	4.6 ± 1.5	4.7 ± 1. 7	
Blood hemoglobin (g/dL)	*N*	10	11	20	0.042*
	Mean ± SD	9.51 ± 1.63	11.16 ± 1.4	10.18 ± 1.4	
Neutrophils (× 10^9^/L)	*N*	6	8	17	0.375
	Mean ± SD	3.8 ± 2.25	2.81 ± 0.87	3.75 ± 1.64	
Lymphocytes (× 10^9^/L)	*N*	6	8	17	0.189
	Mean ± SD	1.65 ± 0.6	1.94 ± 0.71	1.46 ± 0.54	
S. TSAT (%)	*N*	10	11	20	0.478
	Mean ± SD	32.6 ± 9.57	42.27 ± 15.72	37.25 ± 21.92	
S. Ferritin (ng/mL)	*N*	10	11	20	0.654
	Median	352.1	213.7	261.7	
	Q1–Q3	146.27–408.09	172.26–529.00	106.87–351.41	
ESR (mm)	*N*	9	11	16	0.969
	Mean ± SD	27.44 ± 19.6	29.09 ± 15.94	29.06 ± 16.15	
S. hsCRP (mcg/L)	*N*	9	11	16	0.211
	Mean ± SD	12,822.2 ± 7611	13,218.2 ± 8006.7	9012.5 ± 4920.3	
S. IL-6 (pg/mL)	*N*	9	11	16	0.069
	Mean ± SD	5.58 ± 2.89	3.95 ± 1.12	4.01 ± 1.1	
Malnutrition inflammation score (MIS)	*N*	9	10	18	0.192
	Median	7	5	3	
	Q1–Q3	3.00–12.50	2.75–6.00	2.00–6.00	

### Correlation between the three indices and the different biochemical data

Spearman linear correlation was used to measure the correlation between the studied dental and periodontal indices and different scale chronological, hematological, inflammatory, and nutritional variables. DMFT index was significantly positively correlated with RPI as well as with serum transferrin saturation (TSAT) in the studied group, while it was not significantly linearly correlated with the patients’ ages, durations of dialysis, and any other studied biochemical or hematological variables.

On the other hand, RPI scores were observed to have a positive significant correlation with ages of the patients as well as their BMI and TSAT. VPI showed a significant correlation with serum calcium, but not with other biochemical or hematological variables. Dialysis adequacy, expressed as KT/V, was significantly positively correlated with the VPI, but not the other two scores. The studied inflammatory markers as well as the malnutrition–inflammation score did not show any significant linear correlation with any of the three studied dental and periodontal indices (Table 7).

**Table 7:** Spearman's correlations between dental and periodontal indices and the hematological and inflammatory variables of the studied patients

	*N*		DMFT	VPI	RPI
DMFT	43	*r*_*s*_	–	0.044	0.360*
		*p*	–	0.779	0.018
VPI	43	*r*_*s*_	–	–	0.114
		*p*	–	–	0.467
Age (years)	43	*r*_*s*_	0.055	− 0.101	0.525**
		*p*	0.726	0.518	< 0.001
BMI (kg/m^2^)	37	*r*_*s*_	− 0.146	− 0.081	0.438**
		*p*	0.388	0.632	0.007
Dialysis duration (months)	41	*r*_*s*_	0.146	− 0.249	− 0.102
		*p*	0.362	0.116	0.526
KT/V	41	*r*_*s*_	0.255	0.469**	− 0.061
		*p*	0.108	0.002	0.703
Hemoglobin (g/dL)	41	*r*_*s*_	0.181	0.251	0.034
		*p*	0.257	0.113	0.831
Neutrophils (× 10^9^/L)	31	*r*_*s*_	− 0.194	− 0.200	− 0.011
		*p*	0.296	0.281	0.953
Lymphocytes (× 10^9^/L)	31	*r*_*s*_	− 0.101	0.221	0.002
		*p*	0.589	0.233	0.992
S. TSAT (%)	41	*r*_*s*_	0.390*	0.054	0.332*
		*p*	0.012	0.738	0.034
S. ferritin (ng/mL)	41	*r*_*s*_	− 0.232	− 0.108	− 0.148
		*p*	0.144	0.501	0.356
ESR (mm)	36	*r*_*s*_	− 0.089	− 0.097	0.131
		*p*	0.604	0.572	0.445
S. HsCRP (mcg/L)	36	*r*_*s*_	0.086	0.074	− 0.021
		*p*	0.617	0.669	0.902
S. IL-6 (pg/mL)	36	*r*_*s*_	− 0.128	− 0.089	− 0.248
		*p*	0.457	0.608	0.145
Malnutrition inflammation score (MIS)	37	*r*_*s*_	− 0.095	− 0.082	− 0.246
		*p*	0.577	0.629	0.141

^*^Correlation is significant at the 0.05 level (2-tailed)

^**^Correlation is significant at the 0.01 level (2-tailed)

## Discussion

The rising number of patients with ESRD and their treatment is of significant interest to the dental profession, since ESRD is associated with different oral and dental diseases [6, 9, 11, 32]. The majority of studies conducted on ESRD patients have focused only on screening the patients after kidney transplantation without addressing the relation between the oral, systemic, and biochemical parameters [4, 9, 24]. Therefore, the present study aimed to identify the frequency and severity of dental and periodontal problems in patients suffering from ESRD treated by regular hemodialysis, and to explore its clinical and biochemical associations.

The pooled sample of the current study included 43 ESRD patients treated with regular hemodialysis. Our data revealed that males with ESRD were more predominant than females, an observation that matched the results obtained by previous research [26, 33]. An explanation of male preponderance in hemodialysis patients has been attributed to more susceptibility of males to develop CKD and progression to ESRD than females [34]. Furthermore, diabetic males have a higher risk of renal failure than diabetic females [35]. The majority of our patients were of older age with a mean age of 51 years which does not differ from the corresponding values in many previous studies of comparable design [23, 26, 36].

The correlation between the severity of oral diseases and the duration of hemodialysis is still under debate. Previous studies reported the lack of a significant association between hemodialysis duration and the severity of periodontal diseases. It would be reasonable to expect a negative impact of the duration of dialysis on inflammation, immunity, and consequently oral health. However, recent advances in dialysis technology and medical care might mitigate such negative effects. This discrepancy may be due to the differences between patients in various factors including age, diabetes, and smoking status [37]. Furthermore, the higher prevalence of oral diseases among CKD patients may be related to the longer pre-dialysis period with CKD. In addition, negligence of oral hygiene, and the uremic conditions of the patients might have an additional impact [38].

It was hypothesized that diabetic patients on regular hemodialysis showed a higher risk of periodontal diseases in comparison to nondiabetic patients. We detected around one-fifth of our patients were diabetic. Many patients on regular hemodialysis have been receiving treatment for diabetes mellitus for a long time. In addition, diabetes mellitus is considered one of the major causes of periodontal diseases [39, 40]. More than 80% of our cases were hypertensive. It is conceivable that hypertension can be a sequela rather than a cause of renal diseases, at least in a good proportion of cases, explaining the exaggerated prevalence of hypertension in hemodialysis patients [41].

In the current study, more than one-third of the patients had higher serum calcium levels, while only 7% had lower than the target level. In comparison, De Souza et al. [42] reported a calcium mean value of 9 mg/dL and a phosphorus mean value of 5.9 mg/dL, slightly higher than that of the current study. In another previous research by Chloewa et al. [38] the mean values of serum calcium and phosphorus were 8.18 and 6.76 mg/dL, respectively. The reasons for such differences between various studies may be related to dietary factors, dialysis-related factors, and/or the pharmacological use of calcium and phosphate binders. PTH levels play important roles in pathogenesis and progression of hemodialysis-related complications. The median serum level of PTH in the current study was 530 pg/mL, with 25% of values above 950 pg/mL. This finding is similar to previous studies [38, 42].These high level of PTH reflects a widespread prevalence of secondary hyperparathyroidism in hemodialysis population with the association of skeletal and extra-skeletal problems [43].

Strong evidence of an intimate link between dental diseases and advanced ESRD conditions has been reported. Thus, a thorough intraoral examination should be carried out in patients suffering from ESRD. To accurately characterize the descriptive phenomena of the oral examination, some indices were utilized. In the present study, three previously validated indices were adopted; one describes the extension and prevalence of caries (DMFT index), VPI for measuring the oral hygiene of the participants, and a third one measures the extent and severity of periodontal disease (RPI).

In the current study, only about 25% of the patients had a complete set of teeth, while more than a third of the patients had between 1 and 5 missing teeth and around 16% had missing 16 or more teeth. Patients with a complete set of healthy teeth in the present study (*n* = 7) tended to be statistically significantly younger than the others, and this is easily explainable since the rate of teeth decay and extraction occurs more often with increasing age. The previous observation might also be ascribed to age-related increasing incidence and/or progression of some systemic diseases affecting the periodontium, such as diabetes, leading to the loosening of teeth [44, 45].

Teeth filling was more observable in younger patients with a significantly lower mean age of the group of patients with filling teeth. It might be tempting to speculate that younger patients could have the motivation and ability to attend the dental service more than the older ones, thus explaining this difference. The mean DMFT index of the currently studied patients was 5.19 and this is less than previously reported results (11.77) [46]. This discrepancy could be attributed to the worldwide variability of caries prevalence among different countries [47, 48]. The current study reveals a significant positive association between the DMFT index and hepatitis C positivity, where the mean value of the DMFT index was higher in HCV patients than that of the others. This finding matches a previous report of higher DMFT in HCV patients in comparison with healthy controls and this was attributed to the possibly altered immunity and decreased salivary flow in hepatic patients [49].

The correlation between diabetes, ESRD, and the high prevalence of oral diseases is well established, while both diseases can increase the susceptibility to various oral diseases [11, 32, 50, 51].

The mean VPI in the current research was 1.62. Unexpectedly, the mean VPI for nondiabetic patients was significantly more than that of those having diabetes. This might reflect the keenness of some diabetic patients to follow medical advice toward practicing better oral hygiene. There was a significant positive association between RPI and the ages of the participants, a finding which is in harmony with some previous studies [26, 52], while it disagrees with the results of Kshirsagar et al. [53]. It may be possible that both oral hygiene measures and oral care are performed less by older patients than by younger ones. In addition, older people have lower motivation and capability to visit the dentist for routine examinations compared to younger persons.

While obesity is more pronounced in the current work, we detected a significant positive association between RPI and BMI. The association between periodontitis and obesity was confirmed by the study of Nishida et al. [54] which reported that obesity is the second most important independent risk indicator for periodontitis. Furthermore, RPI scores were significantly positively associated with HCV positivity. This matches of Coates et al. [49] who reported more gingival bleeding and deeper pockets in HCV-positive patients. This observation could be attributed to a variety of causes: insulin resistance which is linked to HCV [55], impaired immunity, and persistent chronic inflammation (defect in adherence ability and phagocytosis of neutrophiles which cause a shift in oral bacterial species) [56] in addition to lack of proper dental care in this group of patients.

Several studies have reported no significant correlation between periodontal diseases and serum PTH levels in hemodialysis patients, a finding that aligns with the results of our study [37, 38, 57]. In addition, alveolar bone loss was not correlated to serum PTH level in 35 hemodialysis patients with secondary hyperparathyroidism [57]. Finally, the authors speculated that secondary hyper-hyperparathyroidism and increased serum PTH levels played minimal roles in periodontal disease in hemodialysis patients, a speculation that we also find plausible.

IL-6 is a lymphocyte chemoattractant factor that can be secreted by a wide variety of cells. It is an important cytokine that is among a cascade of mediators released from activated mononuclear cells and a potent mediator of inflammation. As a reaction to the presence of bacteria in periodontal diseases, a variety of immune cells such as monocyte and dendritic cells are activated to phagocytose bacteria and produce inflammatory mediators including IL-6 [58]. In the current study, in contradiction to expectation, there was no significant association between serum IL-6 and RPI. However, an interventional study by D’Aiuto et al. [59] revealed that treatment of periodontal disease decreased serum levels of IL-6. Moreover, it was reported that IL-6 plays a key role in the pathogenesis of periodontal diseases by inducing the differentiation pf osteoclast and bone resorption [60, 61]. In addition, patients with severe forms of apical periodontitis (grades 2 & 3) showed a significant increase in the levels Il-6 in comparison to grade 1 apical periodontitis [62].

Analysis of the impact of nutritional status on periodontal disease revealed no significant association between the MIS and RPI. In contrast to the data of the current study, Chen et al. [37] found a significant correlation between the periodontal status and markers of malnutrition and inflammation. Moreover, other studies reported a significantly higher percentage of patients with malnutrition had periodontitis, a finding that was complemented by the observation that there were more frequent cases of malnutrition in the periodontal disease group than in the non-periodontal disease one [63, 64]. The dissimilarity between this finding of the current study and the previous two studies could be attributable to the sparsity of malnourished cases in the present study, as the mean BMI of the patients of this study was much higher than that in the other two studies. The BMI of the current study sample could indicate an obesity problem rather than undernutrition, unlike the other studies where the BMI was in the normal range.

Evaluation of the periodontal health status of our patients revealed that more than 80% of them had periodontitis, and less than a quarter of the patients had chronic gingivitis. These data are in harmony with the corresponding results of the study of Aboubakr et al. [26], who reported that more than three-quarters of their dialysis patients were afflicted by stage III and IV periodontitis. The present data are also in accordance with those of the Taiwanese study of Chaung et al. [65] who measured periodontitis using the community periodontal index for treatment needs and found that only 1 out of their 128 hemodialysis patients was free from periodontal diseases, while around two-thirds of their patients had periodontitis. Additionally, the severity of periodontitis showed a significant association with the presence of diabetes among the currently studied hemodialysis patients. This finding is in harmony with another study that reported an increased periodontal pocket depth among diabetic CKD patients as compared to that in nondiabetic patients [66].

Our study shows several contributions to bridge the gap in the existing knowledge about the correlation of oral health in ESRD patients undergoing hemodialysis and systemic biochemical profiles. We found a significant correlation between the systemic markers and oral health indices such as DMFT index with serum transferrin saturation and VPI with serum calcium levels, which provides an insight into the systemic factors that may affect oral health in patients with systemic diseases. In addition, HCV-positive patients showed higher DMFT and RPI scores, which necessitates further studies to investigate the impact of HCV in the oral health of patients with ESRD. Despite the high prevalence of periodontitis in diabetic patients, our study showed a lower VPI score in diabetic patients which highlights the awareness of oral hygiene in this subgroup.

## Conclusion

Due to the increased prevalence of plaque accumulation and advanced stages of periodontitis in ESRD patients receiving hemodialysis, we recommend close communication between the nephrologist and the dentist for comprehensive management of ESRD patients on regular hemodialysis. Furthermore, careful instructions of the regular oral hygiene measures as well as regular recall and maintenance visits for periodontal patients must be integrated into the follow-up protocol for ESRD patients.

## Limitations

The direct relationship between renal diseases and oral diseases cannot be concluded from the cross-sectional studies, as the evaluation of dental and periodontal problems was performed during a short period. In addition, the lack of control group made it difficult to compare the clinical and biochemical parameters among ESRD patients with the healthy controls. In addition, the small sample size in the current study limits the generalizability of the data collected.

## Data Availability

The data that support the findings of this study are available from the corresponding author upon reasonable request.
